# 

**Published:** 1888-07

**Authors:** 


					﻿1398.
OLD SERIES
VoL XXV
NEW SERIES
Vol. V.—No. 5.^
& 1855 O


JOURNAi
Ife et	>

C^by
vv.F.WESTMORELAND.M Dj
H.V.M.MILLER.M.D.LL.D.
^bushed By james p.harrison&cg
fl?_____________Atlanta,ga. _ Js
SUBSCRIPTION t 12.50 A YEAR.
				

